# Comparison of juvenile and adult myasthenia gravis in a French cohort with focus on thymic histology

**DOI:** 10.1038/s41598-024-63162-0

**Published:** 2024-06-17

**Authors:** Frédérique Truffault, Ludivine Auger, Nadine Dragin, Jean-Thomas Vilquin, Elie Fadel, Vincent Thomas de Montpreville, Audrey Mansuet-Lupo, Jean-François Regnard, Marco Alifano, Tarek Sharshar, Anthony Behin, Bruno Eymard, Francis Bolgert, Sophie Demeret, Sonia Berrih-Aknin, Rozen Le Panse

**Affiliations:** 1 Center of Research in Myology, Institute of Myology, INSERM, Sorbonne University, 105, Boulevard de l’Hôpital, 75013 Paris, France; 2 Marie Lannelongue Hospital, Paris Saclay University, Le Plessis-Robinson, France; 3 Department of Pathology, Cochin University Hospital Group, AP-HP, Paris-Descartes University, Paris, France; 4 Anesthesia and Intensive Care Department, GHU Paris Psychiatrie et Neurosciences, Pole Neuro, Sainte‑Anne Hospital, Paris, Institute of Psychiatry and Neurosciences of Paris, INSERM U1266, Université Paris Cité, Paris, France; 5 AP-HP, Referral Center for Neuromuscular Disorders, Institute of Myology, Pitié-Salpêtrière Hospital, AP-HP, Paris, France; 6 Neuro-Intensive Care Unit, Pitié-Salpêtrière Hospital, AP-HP, Sorbonne University, Paris, France

**Keywords:** Autoimmunity, Thymus, Follicular hyperplasia, Thymectomy, Puberty, Age, Autoimmune diseases, Neuromuscular disease

## Abstract

Myasthenia gravis (MG) is an autoimmune disease characterized by muscle fatigability due to acetylcholine receptor (AChR) autoantibodies. To better characterize juvenile MG (JMG), we analyzed 85 pre- and 132 post-pubescent JMG (with a cutoff age of 13) compared to 721 adult MG patients under 40 years old using a French database. Clinical data, anti-AChR antibody titers, thymectomy, and thymic histology were analyzed. The proportion of females was higher in each subgroup. No significant difference in the anti-AChR titers was observed. Interestingly, the proportion of AChR^+^ MG patients was notably lower among adult MG patients aged between 30 and 40 years, at 69.7%, compared to over 82.4% in the other subgroups. Thymic histological data were examined in patients who underwent thymectomy during the year of MG onset. Notably, in pre-JMG, the percentage of thymectomized patients was significantly lower (32.9% compared to more than 42.5% in other subgroups), and the delay to thymectomy was twice as long. We found a positive correlation between anti-AChR antibodies and germinal center grade across patient categories. Additionally, only females, particularly post-JMG patients, exhibited the highest rates of lymphofollicular hyperplasia (95% of cases) and germinal center grade. These findings reveal distinct patterns in JMG patients, particularly regarding thymic follicular hyperplasia, which appears to be exacerbated in females after puberty.

## Introduction

Myasthenia gravis (MG) is an autoimmune disease characterized by weakness and fatigability of skeletal muscles. Symptoms often begin with weakness in the eye muscles, resulting in ptosis and diplopia, which define ocular MG. However, they usually progress into generalized MG affecting muscles involved in facial expression, chewing, swallowing, speaking, and limb movement^1^. MG is mediated by autoantibodies targeting proteins of the neuromuscular junction: mainly the acetylcholine receptor (AChR) in 85–90% of patients^2^, but also the muscle-specific kinase (MuSK)^3^ and the low-density lipoprotein receptor-related protein 4 (LRP4)^4^. MG patient classifications are based on several factors: (1) the autoantibody target, (2) the severity of symptoms, (3) the association with a thymic pathology, and (4) the age at onset. Adult MG patients are traditionally categorized as either early onsetMG (EOMG) or late-onset MG (LOMG) occurring before or after the age of 50^5^. EOMG is more common in females, while LOMG tends to affect more males. LOMG may include thymoma-associated patients, with a similar frequency in both males and females^5^. These distinct groups may exhibit specificities related to MG, such as variations in autoantibodies, symptom severity, and treatment approaches.

EOMG is often characterized by B cells infiltrating the thymus, leading to ectopic germinal center (GC) development (lymphofollicular hyperplasia) for AChR^+^ MG^6^. GCs are sites within lymphoid tissues where B cells undergo proliferation, somatic hypermutation, antigen selection, affinity maturation, and differentiation into plasma cells and memory B cells to generate highly specialized and effective antibodies during an immune response. Autoreactive anti-AChR B cells develop in the MG thymus^7,8^. Patients with thymic follicular hyperplasia typically display elevated serum AChR antibody titers^9^. However, there is no clear correlation between the level of AChR antibodies and the severity of the disease. In LOMG, the thymus typically exhibits age-appropriate atrophy and is less commonly associated with thymic follicular hyperplasia^5,10^. Thymoma-associated MG is due to thymic epithelial cell neoplasms but the adjacent thymic tissue can also display GCs^11^.

Juvenile MG (JMG) corresponds to another distinct group of patients. Patients with MG onset before the age of 18 years account for 10–15% of the MG population^12^. This group of patients does not include neonatal MG affecting newborns and caused by the transfer of antibodies from a mother with MG to her baby during pregnancy. In most cases, neonatal MG is temporary, as the mother’s antibodies are gradually cleared from the baby’s system^13^. There is a wide range of variability in the presentation and severity of symptoms in juveniles, as in adult MG patients. However, JMG patients have a higher rate of spontaneous remission^12^.

To better define JMG, we conducted an analysis of data gathered from our French database spanning 40 years, alongside a comprehensive literature review. This analysis of the disease’s specificities in young patients could lead to a better understanding of the etiological mechanisms underlying the disease and, overall, help improve the quality of life for this vulnerable population. Despite the complexity and individualized nature of clinical data within the patient population, resulting in considerable variability across all results, we observed statistically significant findings suggesting a higher susceptibility of females to develop follicular thymic hyperplasia just after puberty.

## Results

### Literature review

Before analyzing the characteristics of JMG patients in our database, we reviewed the literature on JMG cohorts from the past 25 years. Main features are summarized in Table 1. Comparing studies presents a challenge due to inherent differences in patient cohorts. Notable disparities existed across these different studies, such as the number of patients included, ranging from as few as 9 to an extensive cohort of 2161 individuals (Table 1). In addition, differences could be due to “environmental” factors. Indeed, the very high incidence in childhood-onset in China (51.2% of 4219 cases^14^) is attributed to the immunization with live-attenuated Japanese encephalitis vaccine that induces an autoimmune reaction against the AChR through molecular mimicry. We then divided the information contained in Table 1 into two subgroups for Asian and Caucasian populations. Some studies conducted on “Caucasian populations” in Europe or the USA include a certain degree of diversity, with primarily Caucasian patients but also patients of African or Asian origin^15–17^. Of note, some studies underline a high rate of JMG among those of African descent^15,16^.

**Table 1:**
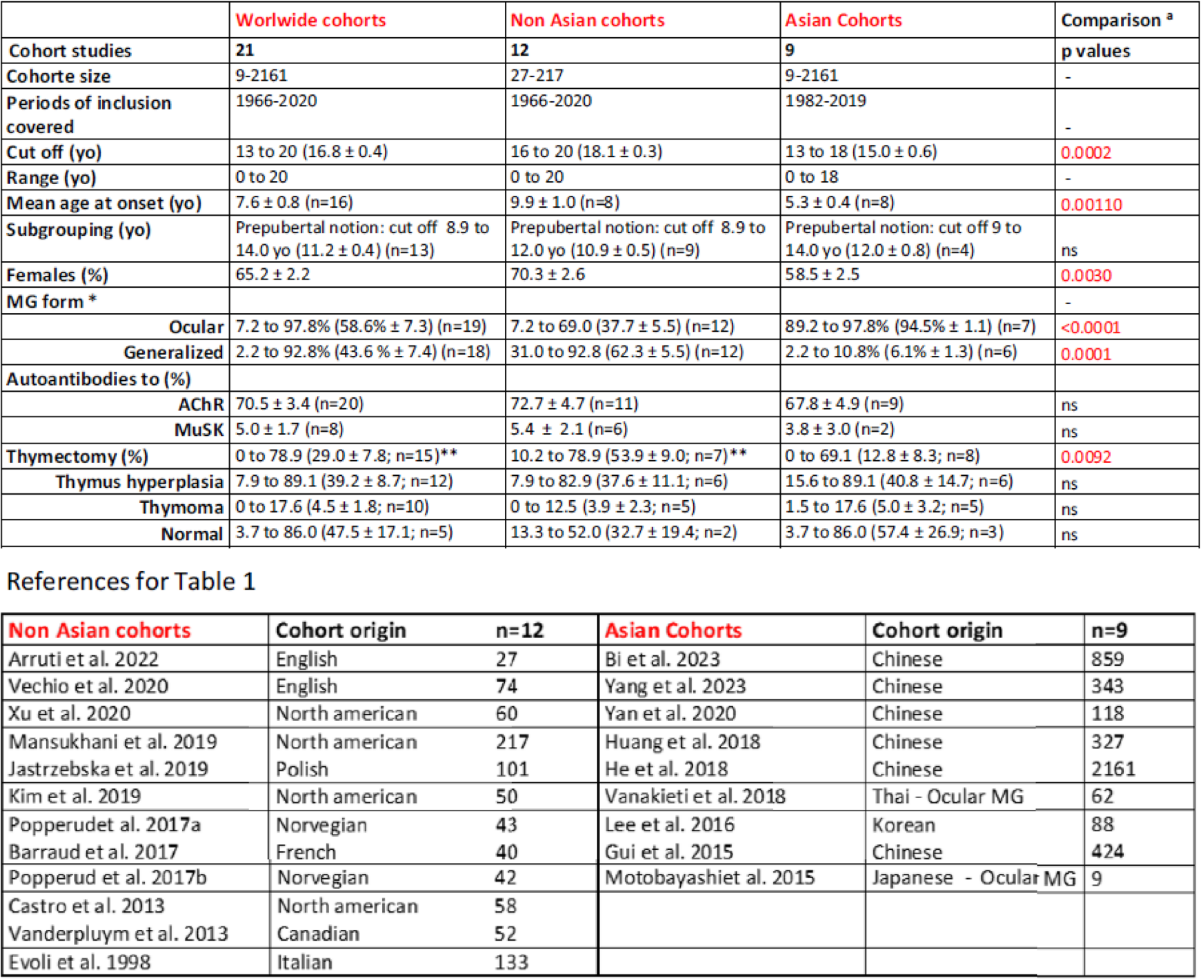
Review of the literature on JMG cohorts.

^a^Comparison of the Asian and non-Asian cohorts.

Mean ± SEM; n: number of studies with the information; yo: years old.

*p*-values calculated with a Mann Whitney test.

*Two studies on pure ocular MG were excluded for the calculation.

**One study on only thymectomized patients was excluded for the calculation.

Comparing these two subgroups, an important factor impacting consistency is the variation in the definition of the age limit for delimiting JMG. Studies define juvenile patients with a cut-off varying from 13 to 20 years old. The mean age of onset is significantly lower in Asian cohorts (5.3 ± 0.4) versus Caucasian cohorts (9.9 ± 1.0). However, it is important to note that this difference could be attributed to variations in the cut-off criteria used to define JMG, which are significantly higher in studies involving Caucasian cohorts (Table 1). Even though there is always a female predisposition in JMG, a significant difference is observed between Caucasian (70.3 ± 2.6) and Asian (58.5 ± 2.5) cohorts. Ocular MG is significantly predominant in Asian JMG, whereas generalized MG is predominant in Caucasian JMG. No differences were observed regarding the proportion of AChR^+^ MG patients in both subgroups (Table 1). We did not compare the treatments and their impacts, but they typically involve a combination of symptomatic and immunosuppressive therapies, with thymectomy in appropriate cases^12,18^. Nevertheless, we analyzed the percentage of thymectomized patients and thymic histology. A higher proportion of JMG are thymectomized in caucasian cohortes.

This analysis highlights significant disparities in patient characteristics depending mainly on the ethnicity of the cohorts. However, we also observed significant variation in the age criteria, which could impact results and may not accurately reflect the characteristics of JMG. (1) In many studies on Caucasian populations, patients under 18 years old are considered as having a juvenile MG. However, an 18-year-old individual may no longer be considered juvenile and rather corresponds to a young adult. (2) Juvenile patients experience the physiological changes of puberty. Some studies substratified JMG patients but this is highly variable across studies. Prepubertal groups were defined with thresholds ranging from 8.9 to 14 years (Table 1). In some studies with very large cohorts, a more important substratification can be proposed such as in the study of Huang et al*.* (327 JMG subdivided into 5 groups: infancy < 1; early childhood < 3; preschool < 6; school < 12; puberty < 18)^19^.

Taking into account these various studies and observations, we conducted an analysis of JMG patients from a French database, comparing them to adult MG patients from the same database.

### Main characteristics of the patient cohort

**Figure 1:**
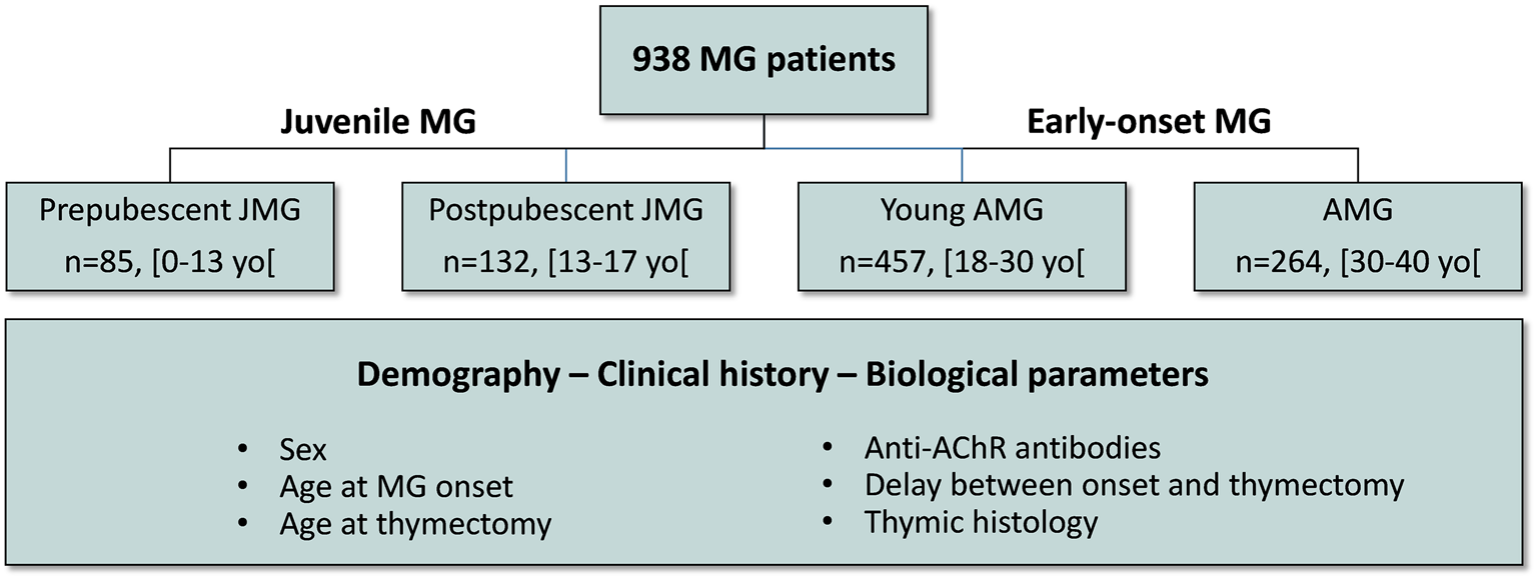
Database data description. Selection of 938 MG patients registered between 1980 and 2019. Patients were stratified into 4 groups: (1) prepubescent JMG under 13 years old (pre-JMG, n = 85), (2) postpubescent JMG aged 13 to 17 (post-JMG, n = 132), (3) young adult MG aged 18 to 29 (YAMG, n = 457), and (4) adult MG aged 30 to 39 (AMG, n = 264). Clinical and biological data including biographic data, anti-AChR antibody titer, thymectomy and thymic histology were analyzed.

To analyze juvenile MG patients, we performed a retrospective analysis of a French database of 938 patients (Fig. 1). We identified 217 JMG below 18 years old that were divided into two groups: prepubescent (pre-JMG, n = 85) and postpubescent JMG (post-JMG, n = 132). Since MG mainly affects women, the cut-off age has been set at 13, as this corresponds to the first menstrual period for women according to the Tanner scale. The cut-off was not obvious to fix because the age of puberty is not the same for males and females. In addition, our database integrates patients between 1980 and 2019, and longitudinal studies over 20 to 30 years have shown that the age of puberty has decreased^20^. The JMG patients were compared to 721 adult MG under 40 years that were also sub-stratified into two groups: Young adult MG (YAMG, n = 457) below 30 years old and an older group aged 30 to 40 (AMG, n = 264) (Fig. 1).

The proportion of MG patients was higher in the YAMG group, which accounted for 48.7% of the patients included in our database, while it was lower for AMG (28.1%), post-JMG (14.1%), and pre-JMG (9.1%) groups (Table 2). The female-to-male sex ratios ranged between 3.0:1 and 4.1:1, with 75.3%, 78.0%, 80.3%, and 77.7% in the pre-JMG, post-JMG, YAMG, and AMG groups, respectively. The highest ratio was observed in YAMG patients compared to the other three groups, but no significant differences were observed (Table 2).

**Table 2:** Characteristics of patients included in the database.

MG cohort (n = 938)		Juvenile MG		Adult MG	
		Before puberty	After puberty	Young	Older
		n = 85	n = 132	n = 457	n = 264
Age range onset		[1–13[	[13–18[	[18–30[	[30–40[
Age at MG onset in years (mean ± SEM)		10.0 ± 2.8	15.3 ± 1.3	23.5 ± 3.3	33.9 ± 2.8
% of patients		**9.1**	14.1	**48.7**	28.1
Females; n (%)		64 (75.3%)	103 (78.0%)	**367 (80.3%)**	205 (77.7%)
MG patients (% for known patients)	AChR positive^a^	70 (82.4%)	**112 (84.8%)**	379 (82.9%)	**184 (69.7%)**
	AChR negative	15 (17.6%)	20 (15.2%)	78 (17.1%)	**80 (30.3%)**
Age at thymectomy in years (mean ± SEM)		15.5 ± 7.7	18.2 ± 5.3	26.5 ± 5.1	36.2 ± 4.0
(n; age range (yo))		(n = 77; 3–56)	(n = 125; 13–49)	(n = 439; 18–51)	(n = 240; 30–53)
Delay between MG onset and thymectomy in months (mean ± SEM)^b^		**64.4 ± 10.6 (n = 77)**	35.9 ± 5.5 (n = 125)	35.7 ± 2.3 (n = 439)	**28.3** ± **2.3 (n** = **240)**

^a^AChR antibodies analyzed with a Fisher’s exact test: AMG vs prepuberty JMG (*p* = 0.0249), postpuberty JMG (*p* = 0.0009) and YAMG (*p* < 0.0001).

^b^Delay between MG onset and thymectomy analyzed with a one-way ANOVA test with Tukey’s multiple comparisons (as detailed in Fig. 3): pre-JMG vs post-JMG (*p* = 0.0012), YAMG (< 0.0001) and AMG (*p* < 0.0001).

Values are shown in bold when they highly differed from those of the other three groups.

**Figure 2:**
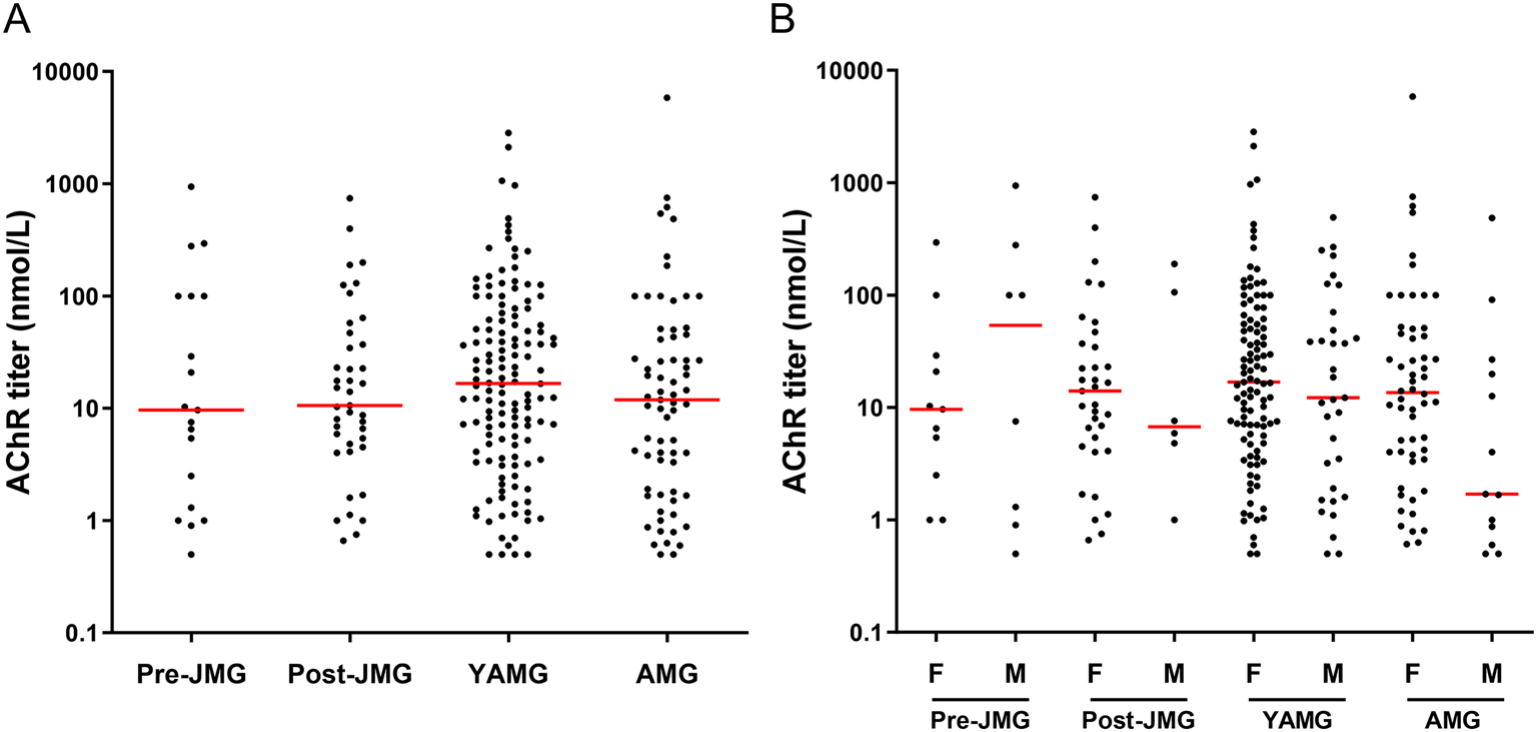
Anti-AChR titers. Anti-AChR titer (≥ 0.5 nmol/L) performed on patients thymectomized the first year of onset were analyzed for 19 pre-JMG, 41 post-JMG, 138 YAMG and 69 AMG (**A**). Analysis of the anti-AChR titer for male and females patients (**B**). Medians are indicated in red. *p*-values were assessed using the one-way ANOVA with Tukey’s multiple comparison tests but no significant differences were found.

The proportion of MG patients with anti-AChR antibodies was significantly lower in AMG group: 69.7% in the AMG group compared to 82.4% (*p* = 0.0249), 84.8% (*p* = 0.0009), and 82.9% (*p* < 0.0001) in the pre-JMG, post-JMG, and YAMG groups, respectively (Table 2). We analyzed the anti-AChR antibody titer for all patients when measured in the year of onset (n = 267). The mean titer was the highest for AMG with 144.6 ± 85.7 nmol/L and the lowest for post-JMG with 58.1 ± 20.8 nmol/L but no significant differences were observed between the different groups (Fig. 2A). In addition, no differences were observed when patients under immunosuppressive treatments were excluded (data not shown). No significant clear differences were observed either if male and female patients were analyzed separately. However, a tendency was observed in the AMG subgroup in which the anti-AChR titers seemed lower in males (mean ± SEM: 49.8 ± 37.1 for males and 166.6 ± 105.1 for females; Fig. 2B).

Regarding the criteria analyzed in this paragraph, they did not highlight very distinctive characteristics for juvenile patients. However, it emphasized a higher proportion of YAMG patients and some slight differences regarding the anti-AChR antibody presence and titer in AMG patients.

### Focus on thymectomy and follicular thymic hyperplasia

**Figure 3:**
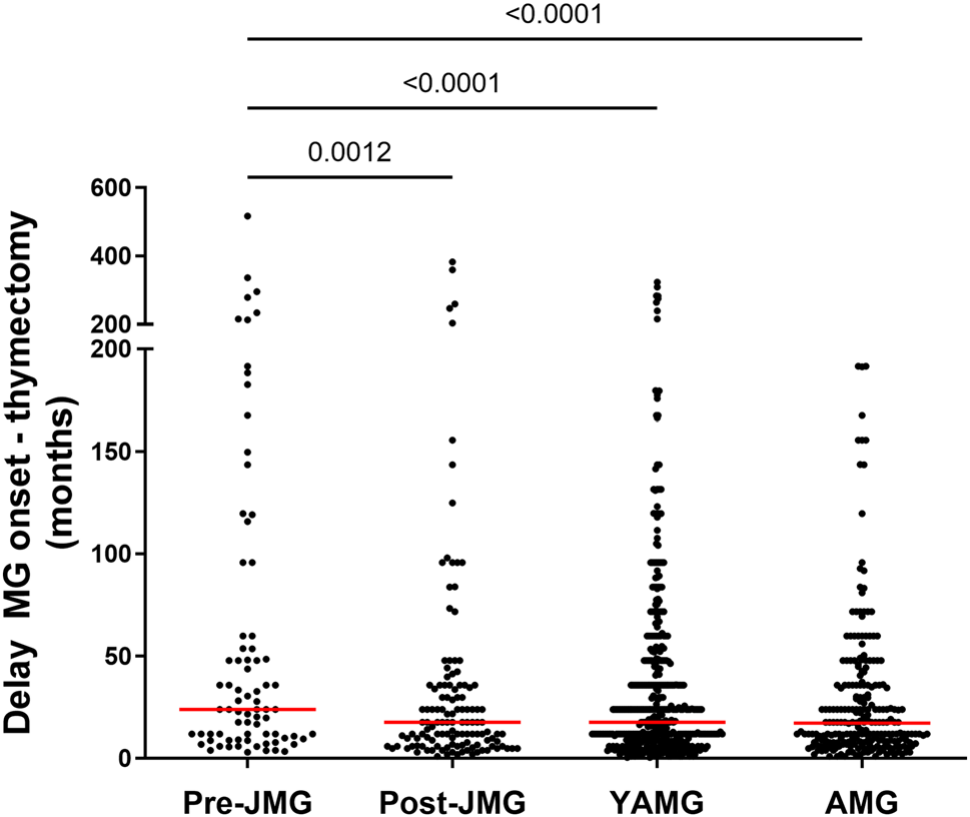
Delay between onset of disease and thymectomy. Time interval between the disease onset and thymectomy in months for 77 pre-JMG, 125 post-JMG, 439 YAMG and 240 AMG. Medians are indicated in red. *p*-values were assessed using the one-way ANOVA with Tukey’s multiple comparison tests and indicated if *p* < 0.05.

In AChR^+^ MG, the thymus is considered as the effector organ and thymectomy leads to progressive improvement of MG symptoms^21^. In our database, the percentage of MG patients thymectomized during the year of onset was significantly lower for pre-JMG (32.9%) as compared to the other groups for which the percentages range between 42.5 and 43.2% (Table 3). Furthermore, we observed that the delay between MG onset and thymectomy was significantly higher for pre-JMG patients than for the other groups. The mean delay for pre-JMG was 64.4 months while they were between 28.3 and 36.0 months for the other groups (Table 2 and Fig. 3). Note that we observed, for each group, that the delay between disease onset and thymectomy was significantly higher for patients who developed the disease before 1980 (data not shown).

**Table 3:** Thymic characteristics for patients thymectomized in the first year after onset number.

MG cohort (n = 393)		Juvenile MG		Adult MG	
		Before Puberty	After Puberty	Young	Older
Age range onset		[1–13[	[13–18[	[18–30[	[30–40[
Thymectomy; n (%)		**28 (32.9%)**	57 (43.2%)	194 (42.5%)	114 (43.2%)
Thymus morphology for all MG patients	Hyperplasia	**16 (80.0%)**	31 (66.0%)	120 (71.9%)	56 (62.2%)
	Normal	4 (20.0%)	16 (34.0%)	47 (28.1%)	34 (31.2%)
	Unknown	8	10	27	24
Presence of thymic GCs in AChR^+^ MG patients without immunosuppressors	Yes^a^	**13 (72.2%)**	**38 (95.0%)**	112 (87,5%)	53 (77.9%)
	None	**5 (27.8%)**	**2 (5.0%)**	16 (12.5%)	15 (22.1%)

^a^Fisher’s exact test for the presence of GCs compared to none: pre-JMG vs post-JMG (*p* = 0.0248), post-JMG vs AMG (*p* = 0.0266).

Values are shown in bold when they highly differed from those of the other three groups.

We analyzed the thymic histological information for patients who were thymectomized in the year of MG onset. Thymic hyperplasia reflecting the enlargement of the thymus gland was more frequently observed in pre-JMG patients but no significant differences were observed between the different groups (Table 3).

**Figure 4:**
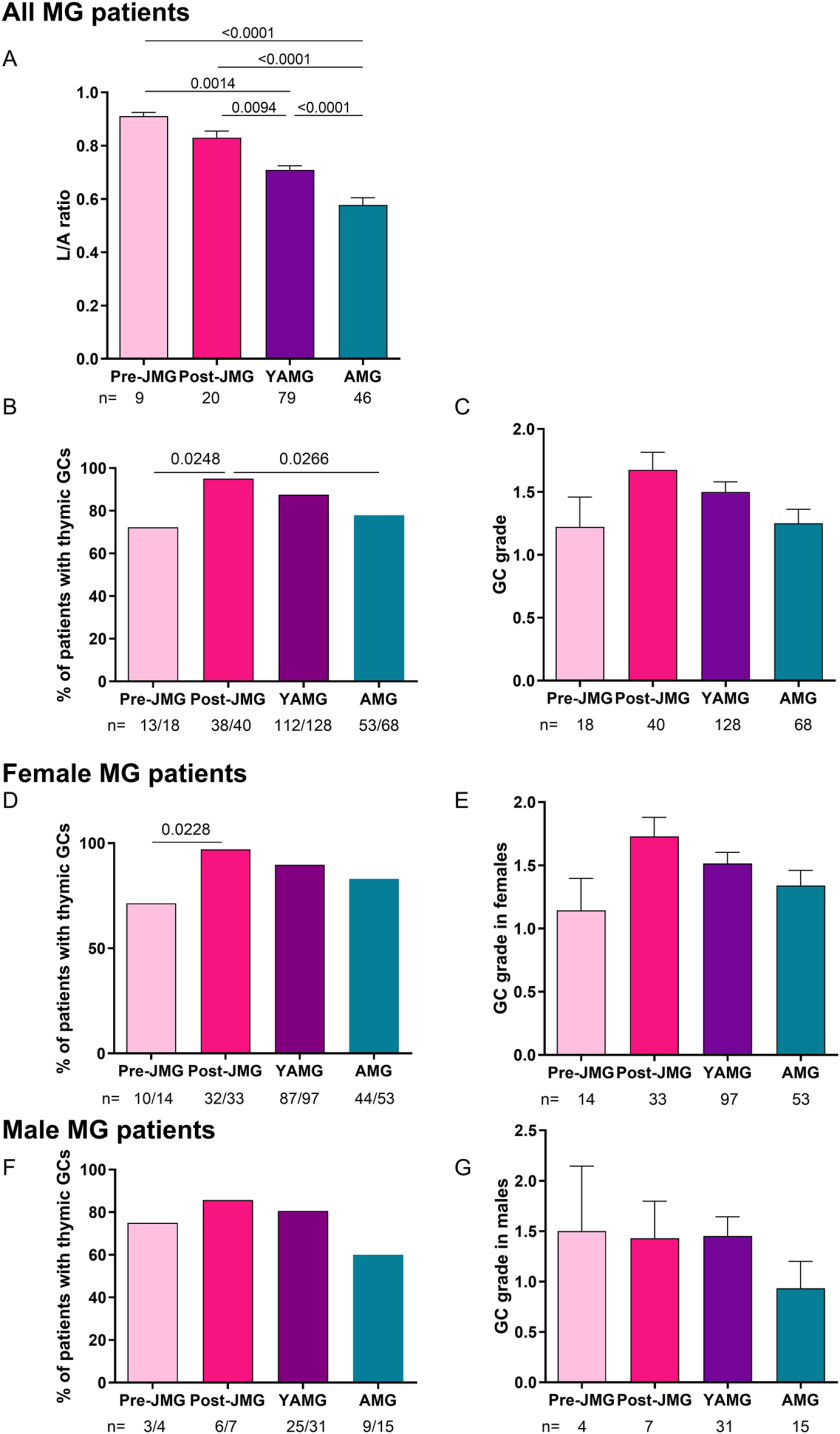
Analyses of thymic histology for the presence of germinal centers. AChR^+^ MG patients without immunosuppressive treatments were analyzed. L/A Lymphocytic/Adipous (L/A) ratio determined for 179 patients by measuring the surface occupied by the lympho-epithelial area among the total tissue (**A**). Percentage of patients with thymic GCs for 296 MG patients (**B**), for 231 female (**D**) or 65 male (**F**) MG patients. The degree of follicular hyperplasia was graded for 296 MG patients (**C**), for 231 female (**E**) or 65 male (**G**) MG patients as follows: no GC = 0; few/rare GCs = 1; many/numerous GCs = 2; very numerous GCs = 3. For figures A, C, E, G graph bars represent the mean values ± SEM. *p*-values were assessed using the one-way ANOVA with Tukey’s multiple comparison tests (A, C, E, G) using a Fisher’s exact test (B-D-F) and indicated if *p* < 0.1.

Next, we only analyzed thymic histological data for AChR^+^ MG patients and patients with immunosuppressive treatments were excluded knowing the impact of corticosteroids on the development of thymic GCs^10,22^ (lower row of Table 3 and Fig. 4). Thymic lymphofollicular hyperplasia is characterized by the development of ectopic GCs. We observed a significant increase in lymphofollicular hyperplasia when comparing post-JMG to pre-JMG and AMG (Table 3).

**Figure 5:**
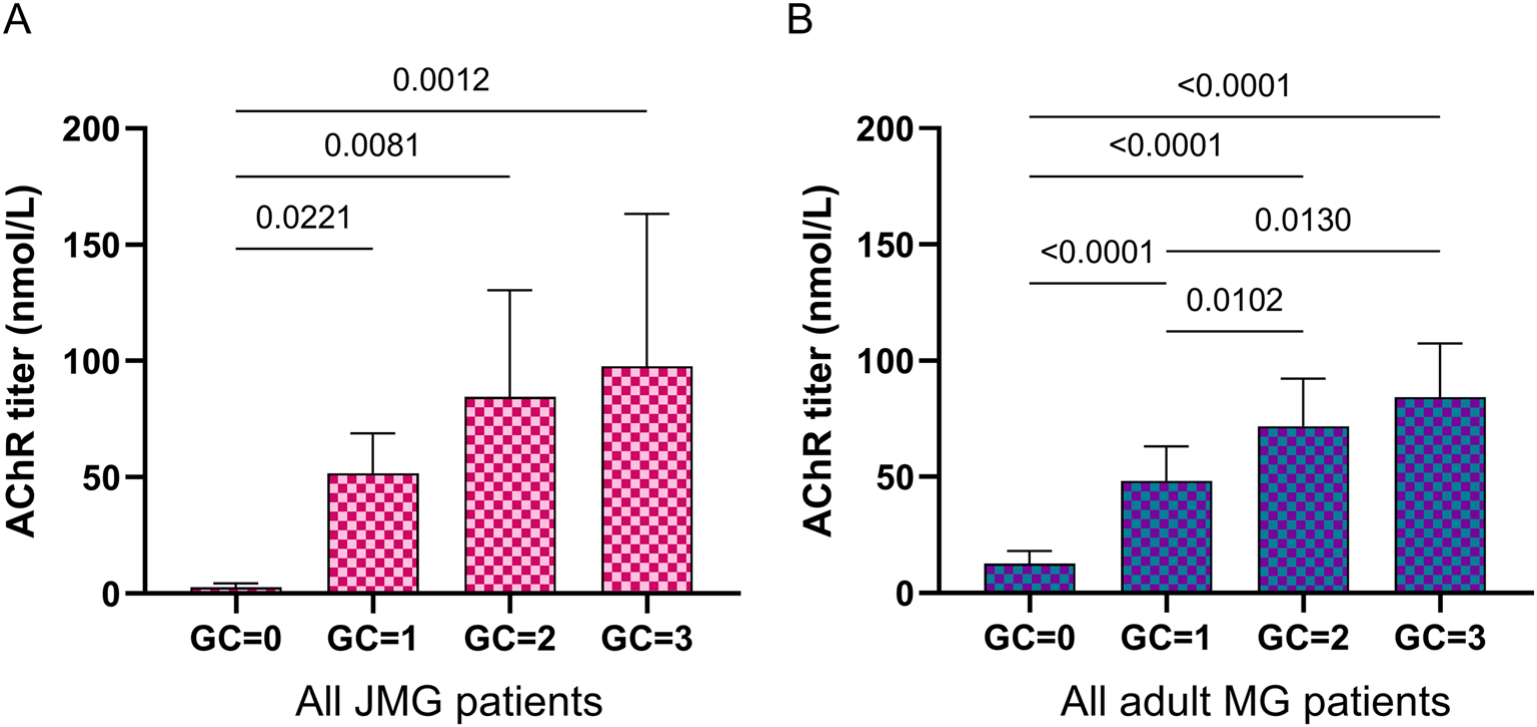
Correlation between the degree of lymphofollicular hyperplasia and anti-AChR titers. AChR^+^ MG patients without immunosuppressive treatments were analyzed. Data from pre- and post-JMG (**A**) and from YAMG and AMG (**B**) were pooled to reach a sufficient number of patients in each category. The degree of follicular hyperplasia was graded as follows: no GC = 0; few GCs = 1; many GCs = 2; numerous GCs = 3. For each grade the mean values ± SEM for the AChR antibodies (nmol/L) was determined. 3 outliers with titers > 2000 nmol/L were excluded to better estimate the correlation. *p*-values were assessed using a Kruskal–Wallis test with Dunn’s multiple comparisons tests (**A**) and indicated if *p* < 0.05.

The thymus is known to involute from puberty, so we analyzed the Lymphocytic/Adipous (L/A) ratio. For all JMG, the L/A ratio was elevated and not significantly different between pre- and post-pubescent patients. However, as expected, the L/A ratio significantly decreased in adults after 18 years old with aging (Fig. 4A). Analyzing the presence of ectopic GCs, we observed again that post-JMG patients have the highest percentage of lymphofollicular hyperplasia (95.0%) (Fig. 4B) associated with the highest grade of GCs (Fig. 4C). This increase in lymphofollicular hyperplasia was observed in females but not in males (Fig. 4D,E). In parallel, in females, the average grade of GCs was higher in post-JMG while in males it was similar in all groups (Fig. 4F,G). We investigated the correlation between anti-AChR antibodies and the grade of GCs. Data from pre- and post-JMG, as well as from YAMG and AMG, were pooled to reach a sufficient number of patients in each category. Our analysis showed a positive correlation between the grade of GCs and the anti-AChR titers for these subcategories (Fig. 5A,B).

Altogether, these data demonstrate that thymic changes were especially observed in females after puberty.

## Discussion

In recent years, there has been a increasing interest in MG patients with disease onset during childhood or before the age of 18 years. Here, we analyzed a French database to better define JMG patients. We stratified JMG patients and compared them to adult patients from the same database. Additionally, we conducted a review of various JMG cohort studies (Table 1) to compare this French JMG cohort with others.

### Comparison of our different subgroups among themselves and with other studies

As pubertal changes can favor the development of autoimmune diseases^23^, we sub-stratified JMG into two groups: pre- and post-pubescent JMG with an age cut-off set at 13 years old. When comparing specifically pre-JMG to post-JMG, we found that the risk of developing MG was lower in individuals under 13 years old. However, there were no significant differences observed in the female-to-male ratios, the percentages of AChR^+^ MG patients, or the anti-AChR titers between the two groups.

To define specific characteristics for JMG patients, our subgroups were compared to adult MG with an early disease onset (EOMG) occurring between the ages of 18 and 45–50. Notably, EOMG patients are not typically stratified. To explore potential differences between young and early midlife EOMG, patients were subdivided into two groups: 18–30 (YAMG) and 30–40 (AMG). Comparison across all four groups revealed that the risk of developing MG was highest between 18 and 30 years old, with nearly half of the patients falling into this category, particularly among females. This sub-stratification also revealed that AMG patients had the lowest percentage of AChR^+^ MG cases. However, there were no significant differences in anti-AChR titers between the groups. In contrast, Andrews et al*.* observed lower levels of AChR antibodies in prepubertal patients^24^.

Compared to other studies, our pre-JMG group shared similarities in age with Asian JMG cohorts, typically defined as under 14 years old. We observed that the mean age at onset was lower in the Asian cohorts. Additionally, our study exhibited a notably high percentage of female JMG patients compared to the average across all studies, including those focusing on Caucasian cohorts. But some studies reported similar or even higher percentages^25–27^. From our analyses, we did not observe critical differences regarding JMG patients, whether pre- or post-JMG, and EOMG patients for the following criteria: the female-to-male ratios, the percentages of AChR+ MG patients, or the anti-AChR titers. We then analyzed the percentage of patients who underwent thymectomy and evaluated thymic histology.

### Thymus implication and thymectomy

A randomized controlled trial of thymectomy in MG adults demonstrates clinical improvement at 3 and 5 years^21^. Similarly, a review of the literature regarding JMG patients indicates improvement following thymectomy^28^. In these cases, patients often require reduced medical therapy, and achieving remission is significant, particularly if thymectomy is performed within two years of onset. However, the impact of thymectomy is less evident in young JMG patients, particularly in Chinese children who often exhibit milder disease and a higher rate of spontaneous remission^12,29^.

Compared to other studies, the notably high incidence of thymectomy in MG patients from our database can be attributed to the fact that our cohort was mainly collected from a specialized cardio-thoracic surgical center. Nevertheless, by comparing our different subgroups, we observed that pre-JMG patients were significantly less often thymectomized in the year of onset, as already observed in 1994 by Andrews et al*.*^24^. Consequently, the delay between onset and thymectomy was significantly higher. The proportion of patients exhibiting thymic hyperplasia was higher in our cohort compared to others, perhaps because thymoma patients were excluded from our analysis. While thymic hyperplasia is often noted across studies, information regarding the presence of GCs (lymphofollicular hyperplasia) is rarely provided^14,30^. GCs are sites of antibody affinity maturation through processes of clonal proliferation, somatic mutation, and selection. They are essentially observed in secondary lymphoid organs but can also be observed in inflammatory organs as in the thymus of AChR^+^ MG patients^31^. Our analysis revealed that the percentage of thymic follicular hyperplasia was the lowest in pre-JMG and increased significantly in post-JMG up to 95%. Lindner et al*.*, in their study of JMG patients aged 13 to 18, a group akin to our post-JMG, reported lymphofollicular hyperplasia in 89% of the patients^30^.

Truffaut et al*.* demonstrated a strong correlation between the degree of follicular hyperplasia and the L/A ratio in MG patients over 20 years old^10^. Similarly, Sarkinen et al*.* observed an inverse correlation between the age at thymectomy and the prevalence of GCs in adult patients^32^. In addition, in adult MG patients, the number of GCs correlates with better post-thymectomy outcomes^32^. Here, among all JMG patients under 18 years old, the L/A ratio was not significantly different but the proportion of patients with a high grade of GCs was higher in post-JMG, especially among females. Additionally, similar to findings in adult MG patients^10,32^, we observed a correlation between anti-AChR titers and the degree of lymphofollicular hyperplasia in JMG. Autoreactive AChR B cells emerge from the thymus and early removal of the thymus could decrease their dispersion in the periphery^8^. Altogether, this suggests that thymectomy could be especially beneficial for female MG patients and should be considered a favorable therapeutic option, especially if performed early in the disease course^28^.

The main changes between the pre- and post-pubescent population are linked to an upheaval in their sexual hormones. These hormones play a crucial role in modulating the formation, function, and maintenance of GCs within lymphoid tissues but also in inflammatory conditions. In GCs, the estrogen receptor α is expressed while the progesterone and androgen receptors are weakly or not expressed. The activation of the estrogen receptor α could favor the development of larger GCs^33^. In contrast, higher serum androgen levels seem to inhibit the development of thymic GCs in MG patients^34^. In addition, through intricate interactions with immune cells and cytokines, sexual hormones influence the processes of B-cell activation, antibody production, and affinity maturation in pathological situations. We previously analyzed the expression by thymic epithelial cells of genes involved in the anti-AChR response in MG, MHC class II and α-AChR subunit, as well as chemokines involved in GC development (CXCL13, CCL21 and CXCL12). We demonstrated that estrogens elicit a consistently low baseline expression of the majority of the analyzed molecules. However, within an inflammatory environment as observed in MG thymus, estrogens provide continuous support for the activation of interferon-signaling pathways, thereby augmenting the progression of GC formation^35^.

### Conclusion

This study is based on data from a specific database representing the French population. Like many retrospective studies covering a long period, it is subject to limitations due to the potential evolution of medical practices over time. In this analysis, we were unable to include MuSK and LRP4 MG patients or account for treatments. However, our study places particular emphasis on the analysis of thymic histology. We showed that postpubescent individuals with JMG have a higher degree of follicular hyperplasia, probably exacerbated by high levels of feminine sexual hormones. Considering that the thymus plays an active role in the production of autoreactive AChR B cells and the correlation between the degree of follicular hyperplasia and the anti-AChR titer^8,9^, this underscores the potential benefits of thymectomy for MG patients before puberty.

## Methods

### Patient database

This study is a retrospective analysis of a computerized and anonymized French database of patients with MG established by Dr. Sonia Berrih-Aknin and her team in collaboration with the Marie Lannelongue Hospital (Le Plessis-Robinson, France). Over the years it has been further implemented with supplementary clinical information from Pitié-Salpêtrière Hospital (Paris, France), Cochin Hospital (Paris, France), and Raymond Poincaré Hospital (Garches, France). 938 MG patients registered from 1980 to 2019 were selected for this study: patients under 40 years old with a precise date of onset (Fig. 1). The diagnosis of MG was mainly based on clinical criteria, detection of AChR antibodies, and electrophysiological study. Patients with a diagnosis of thymoma or thymic carcinoma were excluded.

Patients were stratified into 4 groups defined on the basis of age: (1) prepubescent JMG under 13 years old (pre-JMG, n = 85), (2) postpubescent JMG aged 13 to 17 (post-JMG, n = 132), (3) young adult MG aged 18 to 29 (YAMG, n = 457), and (4) adult MG aged 30 to 39 (AMG, n = 264). The investigations were approved by the local Ethics committee (relevant authorization numbers are ID RCB 2006-A00164-47 and 2010-A00250-39). All analyses were performed in accordance with the relevant guidelines and regulations from these committees, and informed consent was obtained from all participants and/or their legal guardians.

Clinical and biological data including biographic data, anti-AChR antibody titer, thymectomy, and thymic histology were analyzed.

### Thymic histology

MG patients included in our database were mainly thymectomized at the Marie Lannelongue Hospital (Le Plessis-Robinson, France). Nearly all patients were AChR positive. MuSK and LRP4 patients are typically not candidates for thymectomy. Thymic histopathology data were analyzed when thymectomy was performed in the year of disease onset. Several thymic sections were prepared from paraffin-embedded thymic tissues, and stained with hematoxylin–eosin to evaluate the lymphocytic/Adipous (L/A) ratio, and also with an anti-CD20 to complete the analysis for GCs. The degree of follicular hyperplasia was semi-quantitatively graded based on a non-quantitative assessment of the presence of GCs made by different histopathologists: no GC = 0; few/rare GCs = 1; numerous/many GCs = 2; very numerous GCs = 3^10^. Unfortunately, we were unable to employ standardized operating procedures and grading methods, as performed in Germany^36,37^. The lymphocytic/Adipous (L/A) ratio was determined by the surface occupied by the lympho-epithelial area among the total tissue. This value is between 100% (only lympho-epithelial tissue) and 0 (only adipose and connective tissue). This ratio indicates the degree of thymic involution: a high ratio (> 0.7) indicates an abundant lympho-epithelial thymus while a low ratio (< 0.4) indicates a poverty of lymphocytes and the replacement by adipose and connective tissue^10^.

### AChR antibody assay

Anti-AChR antibodies were measured by radioimmunoassay (RIA) mostly at the Necker Hospital (Paris). Patients were considered positive (AChR^+^) from 0.5 nmol/L. Only assays performed on patients thymectomized the first year of onset were analyzed. For AChR-negative (< 0.5 nmol/L) cases, the percentages of MuSK^+^ and LRP4^+^ MG patients were not analyzed. These autoantibodies were discovered in 2001 and 2011, respectively^3,4^, and information was not consistently available for the retrospective cohort spanning over 40 years.

### Statistical analyses

GraphPad Prism 9 was used to perform the statistical analyses and graphic representations. Results were expressed as means ± SEM (standard error to the mean). The Fisher’s exact test was used for contingency table analyses and the one way ANOVA with Tukey’s multiple comparison tests. The tests used were specified in the figure or table captions and *p*-values indicated on graphs if *p* < 0.05.

### Ethics approval and consent to participate

Studies on blood and thymic samples were approved by local ethics committees (RCB 2006-A00164-47 and RCB 2010-A00250-39) and informed consent forms have been collected. The database was registered with the French national data protection authority (CNIL, n°915542).

## Data Availability

Datasets analyzed during the current study are available from the corresponding author on reasonable request.
